# Predicting the Interactome of *Xanthomonas oryzae *pathovar oryzae for target selection and DB service

**DOI:** 10.1186/1471-2105-9-41

**Published:** 2008-01-24

**Authors:** Jeong-Gu Kim, Daeui Park, Byoung-Chul Kim, Seong-Woong Cho, Yeong Tae Kim, Young-Jin Park, Hee Jung Cho, Hyunseok Park, Ki-Bong Kim, Kyong-Oh Yoon, Soo-Jun Park, Byoung-Moo Lee, Jong Bhak

**Affiliations:** 1 Microbial Genetics Division, National Institute of Agricultural Biotechnology (NIAB), Rural Development Administration (RDA), Suwon 441-707, Korea; 2 Korean BioInformation Center (KOBIC), KRIBB, Daejeon 305-806, Korea; 3 Department of Computer Science and Engineering, Ewha Womans University, Seoul 120-750, Korea; 4 Department of Biotechnology and Informatics, Sang Myung University, Cheonan 330-720, Korea; 5 Macrogen Inc., Seoul 153-781, Korea; 6 Bioinformatics Team, Electronics and Telecommunications Research Institute (ETRI), Daejeon 305-350, Korea

## Abstract

**Background:**

Protein-protein interactions (PPIs) play key roles in various cellular functions. In addition, some critical inter-species interactions such as host-pathogen interactions and pathogenicity occur through PPIs. Phytopathogenic bacteria infect hosts through attachment to host tissue, enzyme secretion, exopolysaccharides production, toxins release, iron acquisition, and effector proteins secretion. Many such mechanisms involve some kind of protein-protein interaction in hosts. Our first aim was to predict the whole protein interaction pairs (interactome) of *Xanthomonas oryzae *pathovar oryzae (Xoo) that is an important pathogenic bacterium that causes bacterial blight (BB) in rice. We developed a detection protocol to find possibly interacting proteins in its host using whole genome PPI prediction algorithms. The second aim was to build a DB server and a bioinformatic procedure for finding target proteins in Xoo for developing pesticides that block host-pathogen protein interactions within critical biochemical pathways.

**Description:**

A PPI network in Xoo proteome was predicted by bioinformatics algorithms: PSIMAP, PEIMAP, and iPfam. We present the resultant species specific interaction network and host-pathogen interaction, XooNET. It is a comprehensive predicted initial PPI data for Xoo. XooNET can be used by experimentalists to pick up protein targets for blocking pathological interactions. XooNET uses most of the major types of PPI algorithms. They are: 1) Protein Structural Interactome MAP (PSIMAP), a method using structural domain of SCOP, 2) Protein Experimental Interactome MAP (PEIMAP), a common method using public resources of experimental protein interaction information such as HPRD, BIND, DIP, MINT, IntAct, and BioGrid, and 3) Domain-domain interactions, a method using Pfam domains such as iPfam. Additionally, XooNET provides information on network properties of the Xoo interactome.

**Conclusion:**

XooNET is an open and free public database server for protein interaction information for Xoo. It contains 4,538 proteins and 26,932 possible interactions consisting of 18,503 (PSIMAP), 3,118 (PEIMAP), and 8,938 (iPfam) pairs. In addition, XooNET provides 3,407 possible interaction pairs between two sets of proteins; 141 Xoo proteins that are predicted as membrane proteins and rice proteomes. The resultant interacting partners of a query protein can be easily retrieved by users as well as the interaction networks in graphical web interfaces. XooNET is freely available from .

## Background

Proteins constitute 50 percent or more of the dry weight of living organisms. They have the most diverse biological roles. They function by interacting with other molecules including proteins themselves. Usually, protein-protein interactions are the key mechanisms of normal and pathological functions of living cells. Recently, genomic-scale identification of PPI in model organisms such as *Saccharomyces cerevisiae *[1-3] and *Escherichia coli *[4] have been reported to map the network protein-protein interactions. However, few have been known for phytopathogens and their molecular interactions with hosts. Generally, a phytopathogenic bacterium invades hosts in the following steps: attachment to the host tissue, secretion of degradation enzymes, production of exopolysaccharides, release of toxins, acquisition of iron, and secretion of effector proteins [5]. The gene-for-gene theory that PPI between an effector protein from pathogen and the specific receptor in plant host results in the hypersensitive response and resistance was proposed by Flor [6]. Rossier et al. [7] proposed a model for the role of *Xanthomonas campestris *pv. vesicatoria Hrp proteins in type III secretion and interaction with its plant hosts. Later, Alegria et al. [8,9] proved that the PPI is critical in Hrp type III and type IV secretion systems of *Xanthomonas axonopodis *pv. citri by yeast two-hybrid experiments. There are a few reports on the PPIs involving the effector protein AvrBs3 of *Xanthomonas campestris *pv. vesicatoria [10,11].

Rice (*Oryza sativa*) is one of the major crops in the world, and bacterial blight (BB) causes a huge yield loss (as high as 50% in severely infested fields [12]). Xoo, the rice pathogen causing BB has been completely sequenced [GenBank: AE013598] [13] and the first report on Xoo PPI by glutathione-bead binding experiments. The study included PPIs of several Hrp proteins [14].

Although there was a report showing that some Xoo insertion mutants of unknown or hypothetical protein genes had shown changed pathogenicity [15], it is a long way to go to find all the proteins and their interactions involved in Xoo's pathogenicity. Also, it is expensive and time-consuming to carry out interaction experiments for the whole organism. This led us to develop XooNET which gives us a guidance in targeting pathogenic proteins and their interactions.

In XooNET, predicted PPI information involving Hrp proteins can give us additional function information. For example, Xa21, the resistance gene of rice, has been reported [16]. However, its corresponding Avr protein is yet to be reported. For this instance, the predicted PPI of Xoo can lead users to the function of the effector proteins and finally the target Avr protein(s). There are some pesticides being registered and used against Xoo. However, they were not developed for specific targets, and hence not very effective. The PPI network information Xoo can help the researchers to detect more specific drug targets and increase the pesticide potency.

## Construction and Content

### PSIMAP-based interactions

4,538 proteins of Xoo were retrieved from NCBI and were aligned with SCOP [17] domains using the PSI-BLAST [18] algorithm with a common expect value (E-value) cut-off of 0.001 [19]. By applying SCOP domain interaction pairs obtained from the PSIMAP [20] based interaction information database, PSIbase [21], 18,503 predicted PPIs were obtained for 1,862 Xoo proteins. This was around 41% of the total Xoo proteins.

### PEIMAP-based interactions

The same 4,538 proteins of Xoo were aligned with proteins in PEIMAP using the BLASTP [18] algorithm with a cut-off of 40% sequence identity and 80% length coverage. The PEIMAP includes PPI information from six popular source databases: DIP (Database of Interacting Proteins) [22], BIND (Biomolecular Interaction Network Database) [23], IntAct (Database system and analysis tools for protein interaction data) [24], MINT (Molecular Interactions Database) [25], HPRD (Human Protein Reference Database) [26], and BioGrid (A general repository for interaction datasets) [27]. By applying PEIMAP interaction pairs, 3,118 predicted PPIs were obtained for 629 Xoo proteins. These PPIs was around 14% of the total Xoo proteins.

### Calculating Interactions based on iPfam

Pfam [28] domains of all the Xoo proteins were aligned with hmmpfam by the cut-off of 0.01 (E-value). By integrating them with Pfam domain interaction pairs from iPfam [29], 8,938 predicted protein-protein interactions were constructed with 1,362 selected proteins comprising approximately 30% of Xoo proteins.

### Selecting High-confidence interactions

As a filter, we used the 'combined score' between any pair of proteins which were predicted by PEIMAP, PSIMAP, and iPfam algorithms. As a result, we selected 684 Xoo proteins participating in 2,494 high-confidence PPIs (> 0.6) that were commonly found in all the three databases encompassing PSIMAP, PEIMAP, and iPfam. Those were further rescaled into the confidence range from 0.0 to 1.0 combining all the scores (these were visualized in the Java applet viewer of a modified Integrator program).

### Predicting PPIs between Xoo and Rice

*Oryza sativa *is known as the sole host of Xoo. Therefore, we added 3,407 PPI interaction predictions between Xoo and rice (*Oryza sativa japonica *and *Oryza sativa indica*). We chose 354 proteins expected to be membrane proteins and extra cellular proteins in Xoo using GO-Slim [30]. With these data and PSIMAP, PEIMAP, and iPfam algorithms, we predicted interactions between Xoo and *Oryza sativa japonica *(1,269/26,887), or *Oryza sativa indica *(18/118). As a result, we predicted that 141 Xoo proteins have 3,407 interaction pairs with rice (PEIMAP:25; PSIMAP:2,266; iPfam:2,124). We evaluated many different thresholds of psi-Blast and hmmpfam for domain assignment, and the most adequate one was 10e-4 for PSIMAP, 40% identity and 70% coverage for PEIMAP, and 10e-2 for iPfam.

## Utility

**Figure 1:**
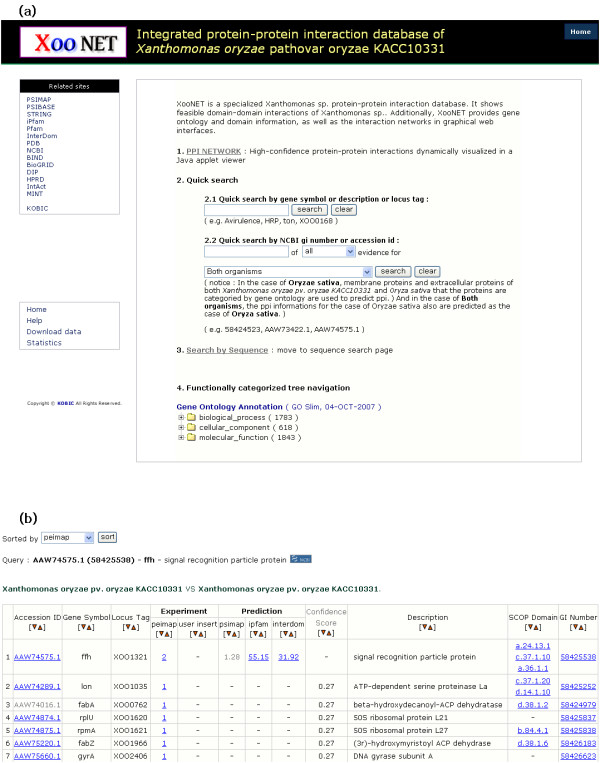
**XooNET system and interfaces**. (a) XooNET integrates four complementary protein-protein interaction databases including PSIMAP, PEIMAP, and iPfam. It shows three search interfaces: (1) search in high-confidence PPI network, (2) keyword and sequence search, and (3) functionally categorized tree navigation of gene ontology annotation. (b) A search result showing the list of predicted interacting proteins, supporting databases, and their synonymous IDs.

XooNET can be accessed by gene symbols, gene descriptions, locus tags, and NCBI gi numbers to find gene information and interacting partners not only of Xoo but also of *Oryza sativa*. Users can also input amino acid sequences. In addition to giving users the functional category of gene sets, XooNET provides the tree of gene ontology annotation using GO-Slim. Figure 1 shows the search interface and the result.

## Discussion

The public interaction databases such as BIND and DIP at this time are limited for the PPIs of Xoo. Therefore, PEIMAP, which is an integrated resource of experimental PPI data, covers only about 14% of the total Xoo proteins. We found that some PPI pairs reported in experiments (Jang et al., 2007; Kim et al., unpublished) were not predicted by XooNET by using the updated PEIMAP algorithm. The cases include: interactions between HrpB1 and RecA, HrpB2 and RecA, HrpB5 and XorII, Hpa2 and RecA; AvrBs2 and HpaP, and AvrBs2 and HrcQ. This shows that the prediction capability of XooNET is still limited for newly discovered protein interactions. By contrast, XooNET predicted that AvrBs2 is interacts with itself. However, a yeast two-hybrid assay showed no self interaction (Kim et al., unpublished). Thus, to increase the prediction boundary of XooNET, we expanded it by providing a field for users to add newly confirmed experimental interaction information.

Avr proteins are known to be crucial effectors that make many bacterial species pathogenic. We found 15 annotated AvrBs3 homologues in Xoo that fall on to three groups according to the interaction promiscuity in protein protein interaction: group 1, zero or one; group 2, more than 60; and group 3, around 10 partners. The highly interactive protein group showed that their numerous partners are functionally related to pathogenicity and can be subdivided. This shows that PPI analysis can assist researchers in discovering new targets and in designing more systematic experiments. One such highly interacting protein, Xoo1125, a hypothetical protein which has over 60 interaction partners including the Avr proteins, caused the loss of pathogenicity when transposon insertion mutation was carried out in a separate experiment. This suggests that XooNET approach is useful in investigating the functions of unknown or hypothetical proteins in *Xanthomonas oryzae *pathovar oryzae.

## Conclusion

XooNET is an integrated database of mutually complementary protein-protein interaction databases: PSIMAP, PEIMAP, and iPfam. The XooNET server is the first specialized Xoo PPI database which provides information of possibly interacting partners against query proteins. In particular, as only one third of the Xoo proteome are fully annotated, there are still many hypothetical and unknown proteins. XooNET provides a platform for biologists to annotate them by predicting their interaction partners and looking into their pathways.

## Methods

### PSIMAP Algorithm

The basic procedure of PSIMAP is to infer interactions between proteins by using their homologs. Interactions among domains or proteins for known PDB (Protein Data Bank) structures are the basis for the prediction. If an unknown protein has a homolog to a domain, PSIMAP assumes that the query tends to interact with its homolog's partners. Its concept is called 'homologous interaction'. The original interaction between two proteins or domains is based on the euclidean distance. Therefore, PSIMAP gives a structure based interaction prediction [20].

### PEIMAP Algorithm

PEIMAP (Protein Experimental Interactome MAP) has been constructed by combining several experimental protein-protein interaction databases. We carried out redundancy check to remove identical protein sequences from the source interaction databases. At present, it contains 116,773 proteins and 229,799 interactions.

## Authors' contributions

JGK started this project, wrote the manuscript, manually validated the interaction lists and tested part of the interaction with yeast two-hybrid system. DP designed the system and wrote the manuscript. BCK constructed the database. SWC developed the website. YTK, YJP and HJC manually validated the web, and performed pathogenicity tests for the Xoo insertion mutants including Xoo1125. HP and KBK constructed automatic graph building modules. KOY and SJP developed pre-search basic modules, gene ontology mapping. BML directed the study. JB conceived and directed the study and helped to draft the manuscript.
